# Differential pathways for the interleukin-1β production activated by chromogranin A and Aβ in microglia

**DOI:** 10.1186/1750-1326-8-S1-P48

**Published:** 2013-09-13

**Authors:** Zhou Wu, Hiroshi Nakanishi

**Affiliations:** 1 Department of Aging Science and Pharmacology, Faculty of Dental Science, Kyushu University, Fukuoka 812-8582, Japan; 2 Japan Science and Technology Agency, Core Research for Evolutional Science and Technology, 5, Sanbancho, Chiyoda-ku, Tokyo 102-0075, Japan

## Background

Although chromogranin A (CGA) is frequently present in Alzheimer’s disease (AD) senile plaques associated with microglial activation, little is known about basic difference between CGA and fibrillar Aβ as neuroinflammatory factors. Here we have thus compared the interleukin-1β (IL-1β) production pathways by CGA and fibrillar Aβ in microglia.

## Materials and methods

MG6 microglia and primary cultured microglia were used in this study. Microglia isolated from young and aged mouse brains by magnetic cell sorting using CD11b-conjugated microbeads were also used. Processings of pro-caspase-1 and pro-IL-1β were analysed by immunoblottings. Secretion of IL-1β was measured by ELISA. The frontal cortex of human brains from AD and no clinical evidence of dementia were used for immunohistochemical analyses.

## Results

In cultured microglia, production of IL-1β was induced by CGA, but not by fibrillar Aβ. CGA activated both nuclear factor-kB (NF-kB) and pro-caspase-1, whereas fibrillar Aβ activated pro-caspase-1 only. For the activation of pro-caspase-1, both CGA and fibrillar Aβ needed the enzymatic activity of cathepsin B (CatB), but only fibrillar Aβ required cytosolic leakage of CatB and the NLRP3 inflammasome activation [1,2]. In contrast, fibrillar Aβ induced the IL-1β secretion from microglia isolated from the aged mouse brain. In AD brain, highly activated microglia, which showed intense immunoreactivity for CatB and IL-1β, surrounded CGA-positive plaques more frequently than Aβ-positive plaques.

## Conclusions

These observations indicate differential pathways for the microglial IL-1β production by CGA and fibrillar Aβ, which may aid in better understanding of pathological significance of neuroinflammation in AD.
